# Epidemiologic profile of inflammatory bowel disease in Eastern Mediterranean Region (EMRO) countries: a systematic review and meta-analysis

**DOI:** 10.1186/s12889-024-18816-z

**Published:** 2024-05-24

**Authors:** Zahra Momayez Sanat, Homayoon Vahedi, Reza Malekzadeh, Zeinab Fanni

**Affiliations:** 1 Assistant Professor of Gastroenterology and Hepatology, Tehran University of Medical Sciences, Tehran, Iran; 2 Digestive Disease Research Institute, Tehran University of Medical Sciences, Tehran, Iran

**Keywords:** Inflammatory bowel disease, Crohn’s disease, Ulcerative colitis, Systematic review, Meta-analysis

## Abstract

**Background:**

Inflammatory bowel disease (IBD) consists of two main types: Crohn’s disease (CD) and ulcerative colitis (UC). The epidemiology of IBD patients has not been comprehensively studied in EMRO countries; therefore, we conducted this meta-analysis to study the epidemiology of this disease in these countries.

**Methods:**

We searched four international databases, namely Scopus, Web of Knowledge (ISI), Medline/PubMed, and ProQuest, from inception up to the end of May 2023. The Preferred Reporting Items for Systematic Review and Meta-analysis (PRISMA) guideline was used to carry out this systematic review and meta-analysis investigation. Using the Joanna Briggs Institute (JBI) Critical Appraisal Checklist, the quality of the selected papers was assessed.

**Results:**

Based on the results of this study, the incidence of UC in EMRO countries was 2.65 per 100,000 (95% CI: 1.39–3.90), and the incidence of CD was 1.16 per 100,000 (95% CI: 0.73–1.59). The most commonly involved intestinal segment in CD was the terminal ileum (44.7%, 95% CI: 34.7–55.2), followed by the ileum (29.8%, 95% CI: 22.2–38.6), and colon (18.7%, 95% CI: 10.8–30.4). However, in UC patients, extensive colitis was the most common finding (32.3%, 95% CI: 26.4–38.8), followed by proctosigmoiditis (27.9%, 95% CI: 21.1–35.8), left-sided colitis (27.4%, 95% CI: 22.7–32.7), and proctitis (22.6%, 95% CI: 17.5–28.5).

**Conclusion:**

As a result, we were able to establish the traits of IBD patients in EMRO nations. UC patients had a higher incidence than CD patients. The most common regions of involvement in CD and UC patients, respectively, were the colon and pancolitis. Compared to UC patients, CD patients had a higher history of appendectomy.

**Supplementary Information:**

The online version contains supplementary material available at 10.1186/s12889-024-18816-z.

## Introduction

Inflammatory bowel disease (IBD) has two main subtypes contains ulcerative colitis (UC) and Crohn’s disease (CD). This disease is becoming a global concern with increasing prevalence and incidence worldwide [1]. Like other Gastrointestinal diseases, IBD has imposed considerable burden globally along with significant population suffering from this condition [2, 3].

Almost 6.8 million cases of IBD were recognized in 2017 globally with the prevalence rate and death rate of 84.3 and 0.51 respectively [4]. It is estimated 2.5 million people in US and 1 million people in Europe suffering from IBD [2]. According to Global Burden of Disease (GBD) statements, North America and Caribbean were the countries with the highest and lowest prevalence of IBD respectively [4]. A study in the UK revealed that the prevalence of IBD has raised 33.8% between 2006 and 2016 [5]. A time-trend analysis has shown that 75% of CD surveys and 60% of UC studies demonstrated a statistically significant growing incidence [6]. In addition, in a study conducted by Caviglia et al., the incidence of IBD was increased from 200 per 100,000 in 2006 to 321.2 per 100,000 in 2021 presenting an increased rate of 46 percent [7].

IBD may occur as a result of the uncontrolled immune system response, which can originate from genetic or environmental determinants [8]. Environmental factors and hereditary susceptibility are the most important cause of the IBD and its course. These two factors arouse the immune system to act over­active and impaired [9, 10]. Smoking, low physical activity, hygiene status, surgeries, and antibiotic consumption are some environmental factors associated with IBD [11]. Based on the epidemiological models, environmental factors can affect individuals based on a person’s genetic characteristics, including age, gender, personality, and physical state, causing IBD susceptibility [10, 12].

Eastern Mediterranean Regional Office (EMRO) includes 22 countries which is one of the World Health Organization regional classifications [13]. The epidemiology of IBD was studies in the EMRO countries separately but a comprehensive study to assess IBD epidemiology was lacking hence we performed a comprehensive meta-analysis study to investigated epidemiological status of IBD in this region.

## Materials and methods

### Setting

The goal of the present research project is to determine the epidemiology of IBD in the EMRO nations by a systematic review and meta-analysis. The Systematic Review and Meta-analysis (PRISMA) protocol was used for executing the study [14].

### Search strategy

We searched four international databases, namely Scopus, Web of Knowledge (ISI), Medline/PubMed, and ProQuest, from inception up to the end of May 2023. The search strategy and keywords are presented in Table 1.

**Table 1:** Search strategy and keywords of this systematic review and meta-analysis

Search query	Keywords (searched through titles, abstracts, medical subject heading (MeSH), affiliations, and general keywords)
Query 1	("Inflammatory Bowel Disease" OR "Crohn's Enteritis" OR "Regional Enteritis" OR "Crohn's Disease" OR "Granulomatous Enteritis" OR "Ileocolitis" OR "Terminal Ileitis" OR "Idiopathic Proctocolitis" OR "Ulcerative Colitis" OR "Colitis Gravis")
Query 2	("Epidemiology" OR "Prevalence" OR "Incidence" OR "Frequency" OR "Risk factor" OR "Risk factors" OR "Related factor" OR "Related factors" OR "Relate factor" OR "occurrence" OR "Associated" OR "associated factor" OR "Odds ratio" OR "Epidemiologic")
Query 3	("Iran" OR "Afghanistan" OR "Bahrain" OR "Djibouti" OR "Egypt" OR "Iraq" OR "Jordan" OR "Kuwait" OR "Lebanon" OR "Libya" OR "Morocco" OR "Oman" OR "Pakistan" OR "State of Palestine" OR "Palestine" OR "Qatar" OR "Saudi Arabia" OR "Somalia" OR "Sudan" OR "Syria" OR "Tunisia" OR "United Arab Emirates" OR "Yemen")
Final search query	Queries 1 AND 2 AND 3

### Inclusion and exclusion criteria

Case–control, cross-sectional, and cohort studies assessing IBD, CD, or UC individuals in the EMRO countries' population with the following criteria were eligible to be included in our study: IBD diagnosis confirmed by clinical characteristics of the individuals and endoscopy or colonoscopy confirmation. At least one of the following outcomes reported: The smoking rate in patients, family history, sites of involvement, risk factors of patients, incidence rate. Studies in English. Available full text. Studies which didn’t fulfill the inclusion criteria were excluded. Two researchers independently selected the studies, and any disagreements were resolved by the third researcher.

### Quality assessment

Using The Joanna Briggs Institute (JBI) Critical Appraisal Checklist, two independent researchers conducted the quality assessment of included cross-sectional, case–control, and cohort articles. Any disagreements were finalized by face-to-face consultation and the contribution of a third researcher. The JBI checklist scores of included studies are shown in Table 2.

**Table 2:** Basic characteristics of included studies

Study name	Country	Study period	Study design	Population	Gender		Sample size			Mean age			Mean age at diagnosis			Level of quality
					Male	Female	UC	CD	IBD	UC	CD	IBD	UC	CD	IBD	
Al Awar, 2004 [15]	UAE	1996–2004	Retrospective study	Pediatric cases with IBD	5	7	8	3	12	4.5	10	NR	NR	NR	NR	Low
El Mouzan, 2012 [16]	Saudi Arabia	1993–2010	Retrospective study	Children below 18 years	111	107	71	147	218	15	16	NR	NR	NR	NR	Medium
Zobeiri, 2017 [17]	Iran	2014–2015	Retrospective study	IBD patients	77	79	153	3	156	NR	NR	31	NR	NR	NR	Medium
Yazdanbod, 2011 [18]	Iran	1998–2008	Retrospective study	patients with UC	44	61	105	-	-	33.5	-	-	NR	-	-	Medium
Siddique, 2014 [19]	Kuwait	2005–2006	Cross-sectional	patients with UC	91	91	182	-	-	NR	NR	NR	28.5	-	-	High
Abdulla, 2017 [20]	Bahrain	1984–2014	Retrospective study	IBD patients	102	85	61	123	187	NR	NR	NR	28.3	24.1	27	Medium
Al-Qabandi, 2011 [21]	Kuwait	1998–2008	Retrospective study	Children with IBD	61	69	36	92	130	NR	NR	NR	NR	NR	10.3	Medium
Al-Mofarreh, 2013 [22]	Saudi Arabia	1993–2009	Retrospective study	IBD patients	387	306	238	455	693	34	27	NR	NR	NR	NR	Medium
Radhakrishnan, 1997 [23]	Oman	1987–1994	Prospective study	patients with UC	52	56	108	17	130	36	NR	NR	NR	NR	NR	Medium
Masoodi, 2012 [24]	Iran	2004–2006	Prospective study	patients with UC	36	43	79	-	-	NR	-	-	32.8	-	-	Medium
Habibi, 2019 [25]	Iran	NR	Cross-sectional	IBD patients	25	36	24	44	71	NR	NR	38.1	NR	NR	NR	Medium
Abdul-Baki, 2007 [26]	Lebanon	2000–2004	Retrospective study	IBD patients	164	87	142	100	251	NR	NR	35.5	32.0	28.8	NR	Medium
Al-Shamali, 2003 [27]	Kuwait	1985–1999	Retrospective study	patients with UC	180	166	346	-	-	NR	NR	45	NR	NR	NR	High
Khan, 1996 [28]	Saudi Arabia	NR	Retrospective study	patients with UC	47	33	80	-	-	36.5	-	-	NR	-	-	Medium
Mirmiran, 2019 [29]	Iran	NR	Cross-sectional	IBD patients	77	66	111	32	143	NR	NR	38.7	NR	NR	NR	Medium
Butt, 2005 [30]	Qatar	1995–2002	Retrospective study	patients with CD	30	20	-	50	-	-	34.2	-	-	NR	-	Medium
Sayar, 2019 [31]	Iran	NR	Cross-sectional	IBD patients	41	19	NR	NR	60	NR	NR	42.2	NR	NR	NR	Low
Vahedi, 2009 [32]	Iran	2004–2007	Retrospective study	IBD patients	205	295	293	207	500	NR	NR	NR	37.1	33.8	NR	Medium
Hosseini, 2015 [33]	Iran	2012–2013	Retrospective study	patients with UC	79	75	154	-	-	42.1	-	-	NR	-	-	Medium
Aljebreen, 2014 [34]	Saudi Arabia	2009–2013	Retrospective study	patients with CD	291	206	-	497	-	-	NR	-	-	25	-	Medium
Ghanadi, 2016 [35]	Iran	2014–2015	Retrospective study	patients with UC	66	84	150	-	-	33.7	-	-	29.4	-	-	High
Fadda, 2012 [36]	Saudi Arabia	1970–2008	Retrospective study	IBD patients	152	160	115	197	312	23.8	28.4	25.5	NR	NR	NR	High
Tavakkoli, 2012 [37]	Iran	2008–2010	Cross-sectional	IBD patients	58	42	70	30	100	NR	NR	NR	NR	NR	34.7	Medium
Shirazi, 2013 [38]	Iran	2005–2007	Cross-sectional	IBD patients	106	94	183	17	200	37.2	32.8	NR	31.5	27	NR	High
El Mouzan, 2006 [39]	Saudi Arabia	1993–2002	Retrospective study	IBD in children < 18 years	19	31	24	19	50	NR	NR	NR	NR	NR	NR	Low
Mansour-Ghanaei, 2015 [40]	Iran	2002–2012	Retrospective study	IBD patients	362	394	756	112	868	46.7	40.1	NR	NR	NR	NR	Medium
Qureshi, 2015 [41]	Pakistan	2008–2012	Cross-sectional	patients with UC	27	27	54	-	-	38.7	-	-	NR	NR	NR	Medium
Ouakaa-Kchaou, 2013 [42]	Tunisia	NR	Cross-sectional	IBD patients	NR	NR	41.5	55.5	202	NR	NR	NR	NR	NR	32	Low
Ghorbaninezhad, 2020 [43]	Iran	2006–2016	Retrospective study	IBD patients	92	98	156	34	190	NR	NR	NR	NR	NR	NR	Low
Balaii, 2015 [44]	Iran	2001–2013	Retrospective study	IBD patients	1120	1137	1914	318	2257	NR	NR	NR	33.8	32.9	NR	High
Al-Ghamdi, 2004 [45]	Saudi Arabia	1983–2002	Retrospective study	patients with CD	33	44	-	77	-	-	25.3	-	-	NR	-	Medium
Esmat, 2014 [46]	Egypt	1995–2009	Retrospective study	IBD patients	79	78	135	22	157	NR	NR	NR	27.3	29.7	NR	High
Alharbi, 2014 [47]	Saudi Arabia	2009–2013	Retrospective study	patients with UC	201	193	394	-	-	30.1	-	-	NR	-	-	Medium
Balaii, 2019 [48]	Iran	2017–2018	Retrospective study	IBD patients	110	137	193	57	259	37.8	36	NR	NR	NR	NR	Medium

*NR* Not reported, *IBD* Inflammatory bowel disease, *CD* Crohn's disease, *UC* Ulcerative colitis

### Data extraction

Included papers were carefully studied by two researchers. The following outcomes were extracted: Name of the first author, year of publication, region of study, duration of study, sample size of study, mean age of participants. The features of included studies are shown in Table 2.

### Statistical analysis

Version 2 of the statistical software for comprehensive meta-analysis (CMA) was employed for this investigation. When three trials were available for a particular outcome, the data were pooled. To ascertain the amount of result heterogeneity, Cochran's test (where the significance level was deemed less than 0.1) and I2 statistics (where the significant level was deemed greater than 50%) were obtained. When heterogeneity was significant, the random-effects model was utilized; otherwise, the fixed-effects model was used.

## Results

**Fig. 1:**
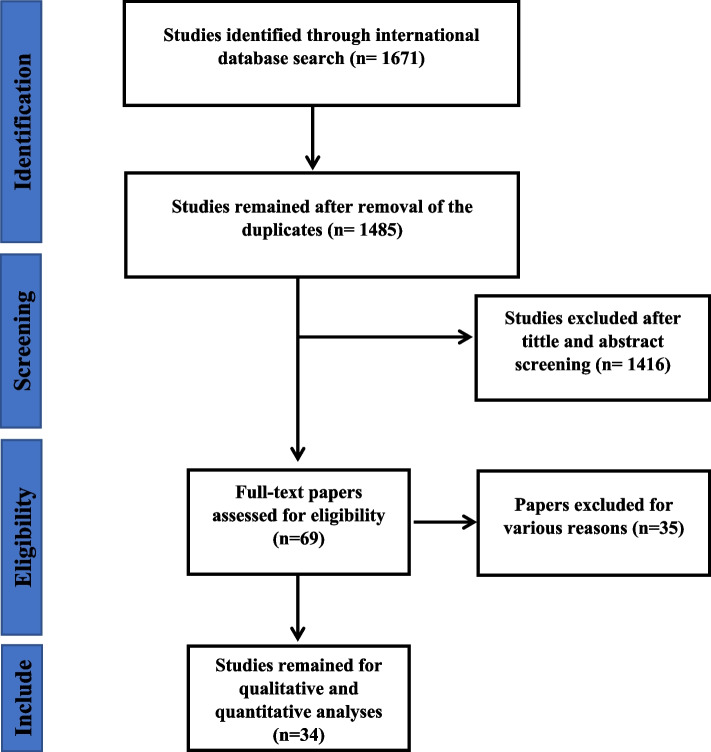
Flowchart of the included eligible studies in systematic review

A total of 1671 studies were found in the initial search. After omitting the duplications, 1485 studies underwent screening. Two researchers independently screened the title, abstract, and, when necessary, the full text of the articles. A total of 1416 articles were deleted, and 69 papers underwent full-text revision. Finally, 34 studies that met our inclusion criteria were selected for our study (Fig. 1).

### Description of studies

The basic characteristics of the included studies are presented in Table 2 [15–48]. Based on the geographical location of the 34 included studies, 14 studies were conducted in Iran, 9 in Saudi Arabia, 3 in Kuwait, 1 in Qatar, 1 in Bahrain, 1 in the UAE, 1 in Lebanon, 1 in Oman, 1 in Pakistan, 1 in Tunisia, and 1 in Egypt. The summary characteristics of the studies are shown in Table 2.

### Incidence of IBD patients

**Fig. 2:**
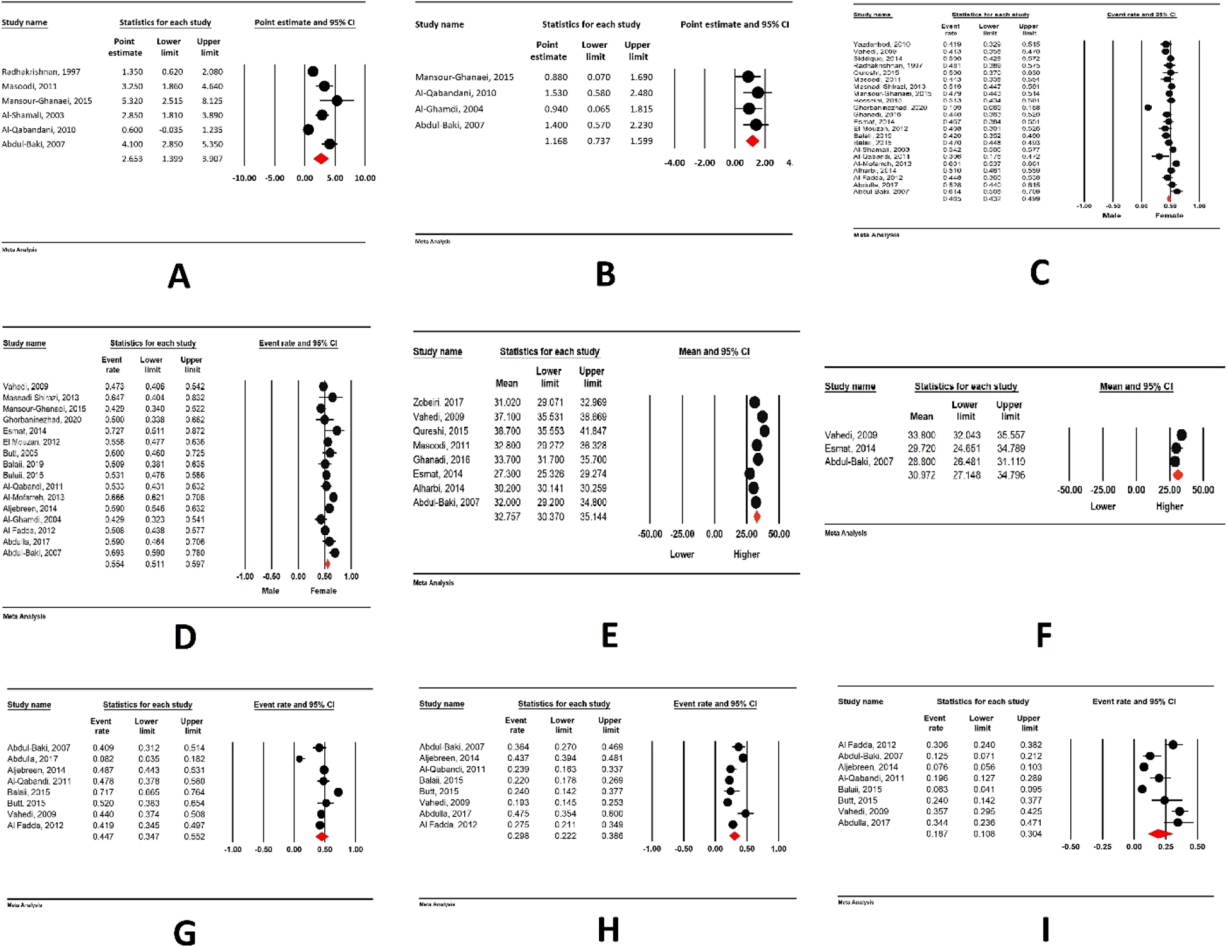
**A** Incidence of UC in EMRO countries, **B** Incidence of UC in EMRO countries, **C** Prevalence of Ulcerative Colitis among men, **D** Prevalence of Crohn Disease among men, **E** Mean Age at diagnosis for Ulcerative Colitis, **F** Mean Age at diagnosis for Crohn Disease, **G** Terminal ileum involvement in CD, **H** Ileal involvement in CD, **I** Colon involvement in CD

According to the results of the meta-analysis, the incidence of UC in EMRO countries was 2.6 per 100,000 (95% CI: 1.3–3.9), and the incidence of CD was 1.16 per 100,000 (95% CI: 0.7–1.5) (Fig. 2A and B).

### Prevalence of IBD among men and women

Based on our meta-analysis, 46% of Ulcerative Colitis diagnoses in EMRO are from men. However, this number is 55% for Crohn Disease (Fig. 2C and D).

### Age at diagnosis

The mean age at diagnosis for Ulcerative Colitis is 32.7 (95% CI: 30.3 to 35.1). In addition, the mean age at diagnosis is 30.9 (95% CI: 27.1 to 34.7) for Crohn Disease (Fig. 2E and F).

### Sites of involvement

**Fig. 3:**
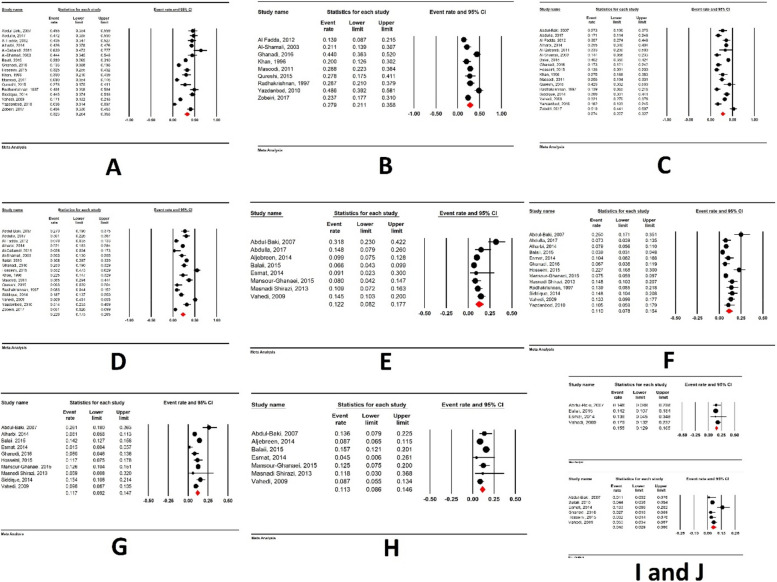
**A** Extensive colitis involvement in UC, **B** Proctosigmoiditis involvement in UC, **C** Left sided colitis involvement in UC patients, **D** Proctitis involvement in UC, **E** Prevalence of smoking in CD patients, **F** Prevalence of smoking in UC patients, **G** Prevalence of positive family history in UC patients, **H** Prevalence of positive family history in CD patients, **I** (Upper figure): History of appendectomy in CD patients, **J** (Lower figure): History of appendectomy in UC patients

The distribution of patients with Crohn's disease (CD) and ulcerative colitis (UC) based on the area of intestinal involvement is depicted in Fig. 2G to I and Fig. 3A to D. In CD patients, the terminal ileum was the most frequently affected intestinal segment (44.7%, 95% CI: 34.7–55.2), followed by the ileum (29.8%, 95% CI: 22.2–38.6), and the colon (18.7%, 95% CI: 10.8–30.4). Regarding UC patients, extensive colitis was the most prevalent finding (32.3%, 95% CI: 26.4–38.8), followed by proctosigmoiditis (27.9%, 95% CI: 21.1–35.8), left-sided colitis (27.4%, 95% CI: 22.7–32.7), and proctitis (22.6%, 95% CI: 17.5–28.5).

### Smoking

The prevalence of smoking in CD patients (12.2%, 95% CI: 8.2–17.7) was higher than in UC patients (11.0%, 95% CI: 7.8–15.4) (Fig. 3E and F).

### Family history

The prevalence of a positive family history in UC and CD was 11.7% (95% CI: 9.2–14.7) and 11.3% (95% CI: 8.6–14.6), respectively (Fig. 3G and H).

### History of appendectomy

The history of appendectomy was higher in CD patients (15.5%, 95% CI: 12.9–18.5) compared to UC (4.8%, 95% CI: 2.9–8) (Fig. 3I and J).

### Result of heterogeneity assessment

As we used random effect model for our main analyses, we presented the detailed information about possible heterogeneity for each outcome in the Table 3. We also evaluated the distribution of true effect using prediction interval (See supplementary material).

**Table 3:** The results of heterogeneity

Variable	Number of included studies	I^2^ (%)	*P*-value	Selected model
Incidence of UC	6	88.1	< 0.001	Random
Incidence of CD	4	0.0	0.658	Random
Terminal ileum involvement in CD	8	92.4	< 0.001	Random
Ileal involvement in CD	8	90.3	< 0.001	Random
Colon involvement in CD	8	94.5	< 0.001	Random
Extensive colitis involvement in UC	17	92.4	< 0.001	Random
Proctosigmoiditis involvement in UC	9	8.9	< 0.001	Random
Left sided colitis involvement in UC	17	90.3	< 0.001	Random
Proctitis involvement in UC	17	92.8	< 0.001	Random
Prevalence of smoking in CD	8	83.7	< 0.001	Random
Prevalence of smoking in UC	13	91.5	< 0.001	Random
Prevalence of positive family history in UC	10	78.3	< 0.001	Random
Prevalence of positive family history in CD	7	51.9	0.052	Random
History of appendectomy in CD	4	0.0	0.700	Random
History of appendectomy in UC	6	79.5	< 0.001	Random

## Discussion

In this study we surveyed the epidemiology of IBD in the EMRO countries. We assessed the incidence of IBD, sites of involvement in GI tract and risk factors.

According to the findings of our study, the incidence rates for UC and CD in the EMRO region were 2.65 and 1.16, respectively. Different nations have distinct rates of incidence and prevalence for IBD and its subtypes. The highest frequency of IBD was found in Europe and North America, according to a comprehensive review and meta-analysis by Ng et al. The incidence of IBD in North America and Europe appeared to be steady or declining based on the findings of this study [1]. The annual incidence rate of CD was reported to be 0.5 per 100,000 in Japan and 20.2 per 100,000 in Canada. In Japan, there were 5.8 UC patients per 100,000 people, compared to 319 UC patients per 100,000 people in Canada [49, 50]. The incidence and prevalence of UC were reported to be 0.3 and 7.6 per 100,000 people in South Korea, respectively [51]. In the United States, prior research places the incidence of UC and CD, respectively, at 10.1 to 12 and 6.3 to 7.9 per 100,000 people [52]. By comparing the findings of our study with those of other studies, we have come to the conclusion that the incidence of UC and CD is higher in the EMRO region than in eastern nations like Japan and South Korea, and lower than in eastern nations. We believe this variation is caused by varying genetic vulnerability, environmental circumstances, and lifestyle choices.

With regard to the findings of our study, CD patients had slightly higher incident rate of smoking (12.2%) than UC patients (11%). In a cohort study conducted by Lunney et al., CD patients had a greater prevalence of smoking than UC patients [53]. Smoking is a difficult component in IBD. Even though it increases the risk of CD, patients with UC benefited from it [54–56]. Smoking’s impact on IBD patients was shown to follow a dosage response pattern [45]. Smoking’s effects on IBD patients can be influenced by genetic and ethnic factors [57, 58].

Positive family history is one of the major risk factors for IBD patients [59]. A person’s genetic and environmental susceptibilities that they inherited from their parents are reflected in their positive family history in IBD patients [60]. First degree relatives and monozygotic twins have a higher incidence of IBD, which supports the hereditary component to IBD [61]. In this study, we demonstrated that UC (11.7%) and CD (11.3%) have slightly higher positive family history rates. Family members of UC patients were much more numerous than CD patients in a meta-analysis research by Childres et al. [62]. Asian, African American, Hispanic, and White populations all had higher rates of positive family history, ranging from 26 to 33%, 9% to 18%, 9% to 16%, and 5.9%, respectively [63–67].

Based to the results obtained in our study, CD patients were more likely to undergo an appendectomy (15.5%) than UC patients (4.8%). Appendectomy's impact on the course of IBD is debatable. According to research by Andersson et al., appendectomy for inflammatory diseases such appendicitis reduces the incidence of UC [68]. Higher risk of CD and UC after appendectomy was found in a different cohort research by Chung et al. [69]. Five years after surgery, an appendectomy significantly reduced the risk of UC in another trial [70].

CD can affect any part of the gastrointestinal tract in a discontinuous manner, whereas UC is limited to the rectum and colon [71]. In this study, we observed that the most common pattern of GI tract involvement in UC patients is extensive colitis (32.3%), followed by proctosigmoiditis (27.9%). For CD patients, the most frequent pattern of involvement was coloileal, followed by the ileum. Previous studies have reported that proctitis and proctosigmoiditis occur in 46% of UC patients, while left-sided colitis and extensive colitis affect 17% and 37% of UC individuals, respectively [72].

### Limitation

Our research had some limitations. First, some of the EMRO region's nations lacked the appropriate literature for our analysis. Second, we do not have adequate data to conduct subgroup analyses based on gender, age, and marital status. Third, we do not have enough information about how many years each patient with IBD has had the disease.

## Conclusions

In conclusion, our study identified the characteristics of patients with inflammatory bowel disease (IBD) in EMRO countries. We observed a higher incidence of ulcerative colitis (UC) compared to Crohn's disease (CD) patients. Coloileal involvement was the most common site of disease in CD patients, whereas extensive colitis was the predominant pattern in UC patients. Additionally, a history of appendectomy was more frequent among CD patients than UC patients.

### Supplementary Information

Supplementary Materials 1.

## Data Availability

No datasets were generated or analysed during the current study.
