# Supplementary material for: Epidemiologic profile of inflammatory bowel disease in Eastern Mediterranean Region (EMRO) countries: a systematic review and meta-analysis
Source: BMC Public Health. 2024 May 24;24:1395. doi: 10.1186/s12889-024-18816-z (PMC11127456; doi:10.1186/s12889-024-18816-z)
Supplement: Supplementary file 1 — Supplementary Materials 1. [file 12889_2024_18816_MOESM1_ESM.docx]

**Supplementary materials**

**Prediction intervals:**

Figure S1: Result of prediction interval based on Arthritis

Figure S2: Result of prediction interval based on colon involvement

Figure S3: Result of prediction interval based on Erythema nodosum

Figure S4: Result of prediction interval based on Eye involvement

Figure S5: Result of prediction interval based on Family history

Figure S6: Result of prediction interval based on History of appendectomy

Figure S7: Result of prediction interval based on Ileum & colon involvement

Figure S8: Result of prediction interval based on Ileum involvement

Figure S9: Result of prediction interval based on Joint pain

Figure S10: Result of prediction interval based on Left-sided colitis

Figure S11: Result of prediction interval based on Oral aphtus

Figure S12: Result of prediction interval based on Pancolitis

Figure S13: Result of prediction interval based on Proctitis

Figure S14: Result of prediction interval based on Proctosigmoiditis

Figure S15: Result of prediction interval based on PSC

Figure S16: Result of prediction interval based on Pyoderma gangrenosum

Figure S17: Result of prediction interval based on Skin involvement

Figure S18: Result of prediction interval based on Smoking
